# Association Between Use of Services To Address Adverse Social Determinants of Health and Documented Suicide Attempt Among Patients in the Veterans Health Administration

**DOI:** 10.1007/s10900-025-01467-5

**Published:** 2025-04-01

**Authors:** John R. Blosnich, Aerin DeRussy, Joshua S. Richman, Melissa E. Dichter, Gala True, Ann Elizabeth Montgomery

**Affiliations:** 1 U.S. Department of Veterans Affairs, Center for Health Equity Research and Promotion, Pittsburgh, PA USA; 2 Suzanne Dworak-Peck School of Social Work, University of Southern California, 669 W 34th Street, Los Angeles, CA 90089 USA; 3 U.S. Department of Veterans Affairs, Birmingham VA Health Care System, Birmingham, AL USA; 4 U.S. Department of Veterans Affairs, Center for Health Equity Research and Promotion, Philadelphia, PA USA; 5 School of Social Work, Temple University, Philadelphia, PA USA; 6 U.S. Department of Veterans Affairs, South Central Mental Illness Research, Education, and Clinical Center, New Orleans, LA USA; 7 Louisiana State University Health Sciences Center-New Orleans, School of Medicine, New Orleans, LA USA; 8 Department of Health Behavior, University of Alabama at Birmingham, School of Public Health, Birmingham, AL USA

**Keywords:** Veterans, Suicide attempt, Social services, Homelessness, Unemployment, Legal problems

## Abstract

Suicide prevention is a top priority for the US Department of Veterans Affairs (VA), and suicide is often associated with adverse social factors (e.g., financial, legal, and housing problems). The VA provides social services integrated with healthcare services, which may increase the opportunities to detect and document suicide attempt in EHR records. Using VA administrative data, we examined three cohorts of all patients from 2014 to 2018 who had housing instability (*n* = 659,987), justice involvement (*n* = 200,487), and unemployment (*n* = 346,556). Administrative records were used to determine ordinal indicators of receipt of VA social services (no services, low, or high). The outcome was suicide attempt noted in the healthcare record (i.e., documented suicide attempt) in the 1–6 months following the incident adverse social factor. We conducted logistic regressions utilizing a discrete-time survival framework with person-month as the unit of analysis, which facilitated accounting for covariates while isolating the independent association of social service utilization. After adjusting for covariates, high receipt of housing services (vs. no services) was significantly associated with documented suicide attempt during the 6-month observation period (aOR = 1.14, 95%CI = 1.06–1.22). A similar association was observed for high vs. no use of justice programs (aOR 1.24; 95% CI:1.12–1.37). There was no significant association between employment services utilization and documented suicide attempt during the 6-month observation period. Our finding that utilization of social services as positively associated with documented suicide attempt likely reflects increased suicide attempt surveillance and documentation with social service involvement. Future research should explore operationalizing patient-level distress in administrative data.

## Introduction

For two decades, the rate of suicide among US military veterans has been higher than the general US population [1]. In 2021, rates of suicide reached their greatest levels of difference: the age-adjusted rate for male veterans was 43% greater than male non-veterans; for female veterans the rate was 166% greater than female non-veterans [1]. Over the decades, these disparities stoked multisectoral initiatives–from community, clinical, and Congressional levels–to reduce suicide among veterans [2], including a devoted crisis line [3], nationwide network of specialized suicide prevention coordinators in all US Department of Veterans Affairs (VA) medical centers [4], and sophisticated risk prediction algorithms with electronic health records (EHR) [5]. Suicide is a complex phenomenon encompassing biopsychosocial factors that are dynamically associated with each other over time, vexing prevention and intervention. While significant efforts focus on biomedically-centered interventions around mental health conditions [6], less research has examined services related to adverse social factors (e.g., lack of employment, housing instability, legal problems) highly associated with suicide risk.

While mental illnesses, primarily depression, are among the strongest predictors of suicide [7], adverse social factors are also strongly associated, proximal risk indicators [8]. For instance, Chen et al. found that among suicide decedents in the National Violent Death Reporting System (NVDRS), 27% had an intimate partner problem [9], which the NVDRS codebook defines as “such as a divorce, break-up, argument, jealousy, conflict, or discord” [10, p.85]. Nearly 12% had a job problem (e.g., “poor performance reviews, increased pressure, feared layoff” or “recently laid off, having difficulty finding a job” ([10], p.115), noted prior to their suicide [9]. Similarly, Dobscha et al. conducted chart reviews of EHR for 269 veterans engaged in VA care and found that 22% had a relationship problem (e.g., “divorce, break-up, arguments or estrangement”, p.S856), which was greater than the prevalence of several mental health diagnoses, such as alcohol use disorder (19.5%) and bipolar disorder (7.3%) [11].

Adverse social factors are also highly associated with suicide attempt. Research from VA EHR found a dose-response-like association between increasing numbers of adverse social factors (e.g., financial instability, legal problems) and odds of suicide attempt [12]. Other studies among veterans have documented independent effects of social adversities such as military sexual trauma (MST) [13] and housing instability with risk of death by suicide [14], and intimate partner violence with increased odds of suicidal ideation and attempt [15]. The strong correlations between adverse social factors and suicide attempt also present opportunities for detection and intervention to prevent further suicidal behaviors or deaths, as previous attempts is one of the strongest predictors of future attempts or death from suicide [16].

Despite robust epidemiologic evidence of adverse social factors’ associations with suicidal ideation, attempted suicide, and death by suicide, theoretical models of suicide have only recently specifically incorporated adverse social factors. For example, the integrated motivational-volitional model of suicide specifically places “life events” as triggering factors and names “social problem-solving” as an example process involved in the motivational phase (i.e., suicidal ideation) [17]. Conversely, the interpersonal theory of suicide posits global constructs of perceived burdensomeness and thwarted belongingness as key factors driving suicidal thoughts and behaviors [18]. Though not named specifically in the interpersonal theory of suicide, adverse social factors can be interpreted as contributing to these constructs (e.g., losing one’s job may contribute to thwarted belongingness and perceived burdensomeness [19]).

Despite the relevance of adverse social determinants, most EHR-based studies predicting suicide attempt focused on biomedical factors (e.g., medical diagnoses, psychotherapy, pharmacotherapy) without accounting for the potential of social service utilization as an independent predictor contributing to the documentation of suicide attempt in HER [5, 20]. The VHA EHR provides a unique opportunity to study the role of social service engagement along with other health factors given the integrated nature of VHA services. Given previous empirical research and theoretical considerations that indicate the importance of adverse social factors in contributing to suicidal thoughts and behaviors [12], it is possible that services to ameliorate adverse social factors, such as housing, employment, and legal problems, may increase the opportunities to detect and document suicide attempt in EHR records. Documenting suicide attempt in the EHR provides opportunities for intervention to prevent future suicide attempt or death from suicide, and strategies of targeting upstream risks for suicide, such as adverse social factors, aligns with calls for public health approaches to suicide prevention [21]. With that perspective, we conducted a retrospective longitudinal study using VA EHR data to test the hypothesis that, among patients with indicators of housing instability, justice involvement, and/or unemployment, receipt of VA services to meet those social needs (i.e., supportive housing, Veterans Justice Outreach [VJO], and vocational rehabilitation) would be positively associated with documented suicide attempt over time.

## Methods

### Data and Analytic Sample

Data were used from several VA administrative sources to build a cohort for the study period of calendar years 2014 through 2018. Structured EHR data came from the VA’s Corporate Data Warehouse (CDW) [22] and additional indicators of housing instability, justice involvement, and unemployment-related information was extracted from the Homeless Operations Management and Evaluation System (HOMES) [23].

Documented suicide attempt was assessed using data from both EHR and the VA Office of Mental Health and Suicide Prevention’s Program Evaluation and Research Center (PERC), which uses data collected by VA’s Suicide Prevention Coordinator network [24]. Details of the data definitions, including International Classification of Disease (ICD)−9/10 codes, stop codes (i.e., VA’s internal coding system for clinical, diagnostic, and therapeutic services), and receipt of VA social services, have been published previously [25].

Across the study period, there were between 6.0 and 6.2 million utilizers of VA care [26]. The foci of the study were patients with one of three types of adverse social factors identifiable due to VA’s robust screening and programming: housing instability, justice involvement, and unemployment [27–29]. The design of this retrospective longitudinal study included a 12-month look-back period during which there were no indications of (i.e., no exposure to) the three adverse social factors—these were referred to as prevalent adverse social factors; the use of a 12-month look-back period to determine prevalent versus incident outcomes is a common timeframe used in VA health services research [30–32]. Incident occurrence of the adverse social factors were identified from the study database from ICD-9/10 codes, affirmation of these social factors during intake assessments, or a clinical screening (in the case of housing instability, as VA implements a universal screening for housing instability [27]).

Three cohorts were constructed representing veterans who experienced each of the three adverse social factors; veterans with more than one adverse social factor were allowed to enter more than one cohort. Veterans with missing or implausible dates of birth, missing or incomplete geographic information, and veterans living outside the 50 United States were excluded. To allow a window of opportunity for all Veterans to have received services to address their indicated adverse social factors, Veterans who died within 30 days of cohort entry or who had a suicide attempt within 30 days of cohort entry were excluded. Follow-up continued for 6 months (divided into intervals of 1 month) from 30 days post-cohort-entry. Follow-up was censored after a Veteran experienced an incident suicide attempt (defined below) or mortality.

### Treatments

Among the three cohorts with indicators of the selected adverse social factors, some patients had records of receiving VA services tailored to address those specific social needs while others had no record of receiving those services. The treatments of interest were receipt of VA social services to address housing instability, justice involvement, and unemployment. Among patients with indicators of at least one of the three adverse social factors, their records were searched to determine if and when they received VA social services for each respective adverse social factor. For the housing instability cohort, services included utilization of any one of several Veterans Health Administration (VHA) Homeless Programs (e.g., Health Care for Homeless Veterans [HCHV]; Domiciliary Care for Homeless Veterans; Grant and Per Diem; US Departments of Housing and Urban Development-Veterans Affairs Supportive Housing [HUD-VASH]; and Supportive Services for Veteran Families). For the cohort with indicators of justice involvement, service utilization included Health Care for Reentry Veterans (HCRV) and VJO. Finally, for the cohort with indicators of unemployment, service utilization included Homeless Veteran Community Employment Services, Compensated Work Therapy, vocational assistance, and employment specialists who were either community-based, through HUD-VASH, or through HCHV. Utilization of services was coded as high (i.e., two or more outpatient visits or one inpatient admission), low (i.e., one outpatient visit), none, or not applicable for patients who did not have an indicator of the adverse social factor.

### Outcome

The primary outcome was documentation of suicide attempt in the 1–6 months following the incident adverse social factor (i.e., the date of entry into each cohort). Suicide attempt documentation included ICD-10 codes and data created by Suicide Prevention Coordinators that are recorded into VA’s Suicide Prevention Applications Network for all nonfatal suicide attempts [33]. This outcome was created as both an ever/never during follow-up variable for descriptive purposes and as a time-varying variable for regression purposes. It is important to emphasize that the outcome is defined as “suicide attempt documentation” because it was based only on evidence from administrative data recorded by providers and did not necessarily reflect patients’ self-reports of attempting suicide.

### Covariates

Several categories of covariates were extracted including demographics of binary sex indicated in the EHR (male, female); age group coded in groups (18–34, 35–44, 45–54, 55–64, *≥* 65); race coded as white, Black, other races, or missing/unknown; ethnicity (Hispanic, non-Hispanic, unknown); marital status coded as married, unmarried, or unknown; patients’ locale (rural, urban); and the region of the country in which the patient resided (northeast, south, midwest, west). Two additional contextual-level indicators were extracted based on patients’ ZIP codes: community poverty level (high, medium, low) and community education level (high, medium, low, missing) [34]. 

Service-connected disability status–a summary rating for patients indicating the extent to which health problems or conditions were caused or worsened by military service – was coded as *≥* 50%, < 50%, no service connection but receiving a VA pension, 0% service connection, or other qualifying status (e.g., prisoner-of-war, Purple Heart recipient). Additional veteran-specific indicators included combat exposure (yes/no) and having reported experience of military sexual trauma (yes/no).

Based on the 24-month lookback period prior to cohort entry, medical diagnoses, mental health diagnoses, and substance use disorder (SUD) diagnoses were extracted and categorized based on the algorithm developed by Elixhauser [35]. A history of suicidal ideation and history of suicide attempt based on ICD-9/10 codes and VA PERC data were also extracted.

Healthcare-related covariates were treated as a time-varying indicators of having received a service or diagnosis since cohort entry, including any emergency department visits (yes/no), and any inpatient admissions for medical (yes/no), mental health (yes/no), or substance use disorder (SUD) (yes/no). Outpatient visits were more common and were summarized for descriptive purposes using the median and interquartile range for the following types of visits: primary care, medical, mental health, and SUD. For regression purposes, the sum of each type of outpatient care received was entered into the model as a continuous variable.

### Analyses

Descriptive statistics were used to summarize all variables in each of the three cohorts. Then bivariate analyses were conducted to compare the characteristics of veterans with and without documented suicide attempt during the follow-up period using chi-square and Wilcoxon signed rank tests, as appropriate.

Logistic regressions were constructed utilizing a discrete-time survival framework with person-month as the unit of analysis and suicide attempt as the outcome. The logistic approach allowed accounting for covariates while isolating the independent association of treatment utilization (i.e., services to address adverse social factors) with documented suicide attempt. The discrete-time survival framework allowed direct modeling of suicide attempt documentation rates during the 1–6 months period, adjusting for calendar time, seasonality, and the inclusion of time-varying predictors [36]. As mentioned above, Veterans who died or attempted suicide in the first 30 days of cohort entry were excluded; person-months beginning at 30 days after cohort entry—in effect, a landmark analysis— were modeled to avoid immortal time bias [37]. Estimates are reported as adjusted odds ratios (aOR) with corresponding 95% confidence intervals (CI), and all analyses were conducted using SAS. This study was approved by the institutional review boards of [institution names redacted for peer review].

## Results

After applying inclusion and exclusion criteria to all VA enrollees during the study period, the three, separate analytic cohorts comprised patients with an indicator of housing instability (*n* = 659,987), patients with an indicator of unemployment (*n* = 346,556), and patients with an indicator of justice involvement (*n* = 200,487). Among the housing instability cohort, approximately 60% of patients received some services during the first 6 months to address housing concerns, and a similar prevalence among the cohort with justice involvement received relevant services (Table 1). Among the unemployment cohort, about 40% of patients received some services to address employment problems.

**Table 1:** Characteristics of study cohorts

	Housing Instability	Unemployment	Justice Involvement
	n (%)	n (%)	n (%)
N	659,987 (100)	346,556 (100)	200,487 (100)
Sex			
Male	585,288 (88.3)	308,283 (89.0)	186,800 (93.2)
Female	74,699 (11.3)	38,273 (11.0)	13,687 (6.8)
Age			
18–34	110,798 (16.7)	63,338 (18.3)	45,782 (22.8)
35–44	80,055 (12.1)	47,409 (13.7)	29,791 (14.9)
45–54	127,709 (19.3)	81,576 (23.5)	42,089 (21.0)
55–64	195,014 (29.4)	104,031 (30.0)	50,014 (25.0)
65+	146,411 (22.1)	50,202 (14.5)	32,811 (16.4)
Race			
White	375,896 (56.7)	198,911 (57.4)	127,291 (63.5)
Black	218,421 (33.0)	117,840 (34.0)	53,480 (26.7)
Another racial category	24,659 (3.7)	11,988 (3.5)	7,079 (3.5)
Missing/unknown	41,011 (6.2)	17,817 (5.1)	12,637 (6.3)
Ethnicity			
Hispanic	45,128 (6.8)	22,824 (6.6)	15,098 (7.5)
Non-Hispanic	588,202 (88.8)	311,993 (90.0)	176,893 (88.2)
Unknown	26,657 (4.0)	11,739 (3.4)	8,496 (4.2)
Marital Status			
Married	172,405 (26.0)	90,859 (26.2)	54,826 (27.4)
Unmarried	476,548 (71.9)	250,472 (72.3)	140,648 (70.2)
Unknown	11,034 (1.7)	5,225 (1.5)	5,013 (2.5)
Eligibility category			
SC > = 50	247,261 (37.3)	137,998 (39.8)	88,065 (43.9)
SC < 50%	128,231 (19.4)	68,394 (19.7)	37,564 (18.7)
Not SC, VA Pension	32,900 (5.0)	16,467 (4.8)	7,896 (3.9)
Not SC	239,305 (36.1)	118,305 (34.1)	64,761 (32.3)
Other	12,290 (1.9)	5,392 (1.6)	2,201 (1.1)
Combat	104,394 (15.8)	62,961 (18.2)	43,858 (21.9)
Military sexual trauma	59,389 (9.0)	34,558 (10.0)	16,700 (8.3)
Geographic location			
Rural	147,405 (22.2)	74,807 (21.6)	51,251 (25.6)
Urban	512,582 (77.4)	271,749 (78.4)	149,236 (74.4)
Region			
Northeast	86,125 (13.0)	44,507 (12.8)	23,964 (12.0)
South	271,294 (41.0)	144,466 (41.7)	81,282 (40.5)
Midwest	120,279 (18.2)	73,650 (21.3)	42,512 (21.2)
West	182,289 (27.5)	83,933 (24.2)	52,729 (26.3)
Community poverty level			
High	174,247 (26.3)	94,831 (27.4)	50,013 (25.0)
Medium	427,923 (64.6)	220,568 (63.7)	131,528 (65.6)
Low	57,817 (8.7)	31,157 (9.0)	18,946 (9.5)
Community education level			
High	79,090 (11.9)	42,388 (12.2)	25,696 (12.8)
Medium	270,386 (40.8)	140,486 (40.5)	84,335 (42.1)
Low	212,455 (32.1)	110,368 (31.9)	60,343 (30.1)
Missing	98,056 (14.8)	53,314 (15.4)	30,113 (15.0)
Inpatient admission			
Medical	55,154 (8.3)	27,721 (8.0)	14,665 (7.3)
Mental health	27,010 (4.1)	17,010 (4.9)	10,548 (5.3)
SUD	27,527 (4.2)	19,568 (5.7)	15,328 (7.7)
Emergency department visits	209,842 (31.7)	119,809 (34.6)	61,490 (30.7)
Outpatient visits (median, IQR)			
Primary care	2 (1–4)	2 (1–4)	2 (0–4)
Medical	3 (1–9)	4 (1–11)	2 (0–9)
Mental health	1 (0–5)	3 (0–9)	2 (0–9)
SUD	0 (0–0)	0 (0–1)	0 (0–3)
Homeless services use			
High	277,543 (41.9)	128,898 (37.2)	59,553 (29.7)
Low	133,983 (20.2)	16,952 (4.9)	9,246 (4.6)
None	248,461 (37.5)	28,748 (8.3)	19,253 (9.6)
N/A (no homeless indicator)	N/A	171,958 (49.6)	112,435 (56.1)
Unemployment services use			
High	38,398 (5.8)	101,842 (29.4)	15,890 (7.9)
Low	18,654 (2.8)	47,208 (13.6)	6,723 (3.3)
None	104,578 (15.8)	197,506 (57.0)	41,605 (20.7)
N/A (no unemployment indicator)	498,357 (75.2)	N/A	136,269 (68.0)
Justice services use			
High	30,708 (4.6)	25,332 (7.3)	71,797 (35.8)
Low	14,249 (2.1)	10,663 (3.1)	48,884 (24.4)
None	29,888 (4.51)	23,033 (6.6)	79,806 (39.8)
N/A (No Justice Indicator)	585,142 (88.3)	287,528 (83.0)	N/A
Any medical diagnosis	326,465 (49.3)	189,377 (54.7)	92,535 (46.2)
Any mental health diagnosis	283,847 (42.8)	199,821 (57.7)	106,610 (53.2)
Any SUD diagnosis	156,830 (23.7)	129,230 (37.3)	78,939 (39.4)
Baseline documented suicide ideation	77,320 (11.7)	60,396 (17.4)	36,655 (18.3)
Baseline documented suicide attempt	22,579 (3.4)	17,692 (5.1)	11,889 (5.9)
Documented suicide ideation	35,311 (5.3)	26,856 (7.8)	13,904 (6.9)
Documented suicide attempt	4,745 (0.7)	3,224 (0.9)	2,142 (1.1)

Notes. SC = service-connected; SUD = substance use disorder; IQR = interquartile range

The housing instability cohort had the lowest baseline prevalence (i.e., prior to observation period) of documented suicide attempt (3.4%) while the justice involvement cohort had the highest (5.9%). This same pattern emerged for incident documented suicide attempt, which ranged from a low of 0.7% among the housing instability cohort to 1.1% for the cohort with justice involvement.

For patients with experience of housing instability, utilization of services to address adverse social factors (i.e., the main effect being studied) was significantly associated with documented suicide attempt during the 6-month observation period. Specifically, after adjusting for covariates, among patients with an indicator of housing instability, high receipt of VHA Homeless Program services was associated with a 1.14 increased odds of documented suicide attempt compared to no receipt of VHA Homeless Program services (Table 2). Low receipt of VHA Homeless Program services was associated with 1.10 greater odds of documented suicide attempt compared to no receipt of VHA Homeless Program services. For patients with an indicator of unemployment, there was not a statistically significant association between use of employment services and documented suicide attempt during the 6-month observation period (Table 3). Finally, patients with an indicator of justice involvement had increased odds of documented suicide attempt (aOR 1.24; 95% CI:1.12–1.37) associated with high use of VHA Justice Program services compared to no receipt of VHA Justice Program services (Table 4). The association between documented suicide attempt and low receipt of VHA Justice Program services was not statistically significant.

**Table 2:** Logistic regression predicting documented suicide attempt among veterans with an Indicator of housing instability, months 1–6 (*N* = 659,987)

Variable	aOR	95% CI		*p*-value
Sex Female (ref male)	0.96	0.87	1.05	0.370
Age (ref 45–54)				
18–34	**1.49**	**1.35**	**1.63**	**< 0.0001**
35–44	1.08	0.98	1.19	0.100
55–64	**0.72**	**0.66**	**0.79**	**< 0.001**
65+	**0.40**	**0.35**	**0.46**	**< 0.001**
Race (ref Black)				
White	**1.63**	**1.51**	**1.76**	**< 0.001**
Another racial category	**1.34**	**1.14**	**1.58**	**0.000**
Missing/unknown	**1.22**	**1.04**	**1.45**	**0.020**
Ethnicity (ref Hispanic)				
Non-Hispanic	1.06	0.95	1.19	0.300
Unknown	1.12	0.91	1.37	0.290
Marital status (ref married)				
Unmarried	0.96	0.90	1.03	0.280
Unknown	1.00	0.78	1.28	1.000
Eligibility category (ref SC > = 50%)				
SC < 50%	**0.82**	**0.76**	**0.90**	**< 0.001**
Not SC	**0.76**	**0.70**	**0.83**	**< 0.001**
Not SC, VA Pension	0.91	0.77	1.06	0.230
Other	1.03	0.79	1.35	0.840
Combat exposure (ref none)	**0.89**	**0.82**	**0.96**	**0.003**
Military sexual trauma (ref none)	**1.44**	**1.32**	**1.57**	**< 0.001**
Rural (ref urban)	0.98	0.91	1.05	0.580
Region (ref West)				
Midwest	**0.89**	**0.82**	**0.97**	**0.010**
Northeast	**0.68**	**0.61**	**0.76**	**< 0.001**
South	**0.85**	**0.79**	**0.91**	**< 0.001**
Community poverty level (ref low)				
High	0.98	0.86	1.11	0.720
Medium	1.01	0.91	1.12	0.880
Community education level (ref low)				
High	1.07	0.96	1.18	0.240
Medium	1.04	0.97	1.12	0.280
Missing	1.04	0.95	1.15	0.400
Inpatient admission				
Medical (ref none)	**1.18**	**1.04**	**1.32**	**0.010**
Mental health (ref none)	**1.45**	**1.31**	**1.60**	**< 0.0010**
SUD (ref none)	**1.51**	**1.36**	**1.67**	**< 0.0010**
Emergency department visit (ref none)	**1.28**	**1.20**	**1.38**	**< 0.0010**
Outpatient visits				
Primary care (n) (ref none)	**0.99**	**0.97**	**1.00**	**0.020**
Medical (n) (ref none)	**0.99**	**0.99**	**1.00**	**< 0.001**
Mental health (n) (ref none)	**1.07**	**1.00**	**1.01**	**< 0.001**
SUD (n) (ref none)	**0.99**	**0.99**	**1.00**	**0.000**
Any medical diagnosis (ref none)	**0.92**	**0.86**	**0.99**	**0.020**
Any mental health diagnosis (ref none)	**1.53**	**1.41**	**1.66**	**< 0.001**
Any SUD diagnosis (ref none)	**1.17**	**1.09**	**1.26**	**< 0.001**
Baseline documented suicide ideation (ref none)	**1.84**	**1.71**	**1.98**	**< 0.001**
Baseline documented suicide attempt (ref none)	**3.19**	**2.96**	**3.44**	**< 0.001**
Documented suicide ideation (ref none)	**2.59**	**2.38**	**2.81**	**< 0.001**
Homeless services use (ref none)				
High	**1.14**	1.06	1.22	0.002
Low	**1.10**	1.01	1.19	0.030
Employment services use (ref N/A no unemployment)				
High	0.99	0.89	1.11	0.920
Low	**1.26**	**1.10**	**1.44**	**0.000**
None	**1.10**	**1.02**	**1.19**	**0.010**
Justice services use (ref N/A no justice involvement)				
High	1.07	0.96	1.19	0.250
Low	**1.20**	**1.04**	**1.39**	**0.010**
None	**1.14**	**1.02**	**1.28**	**0.020**

Notes. Model also controls for person month, calendar month, and calendar year. aOR = adjusted odds ratio; CI = confidence interval; SC = service-connected; SUD = substance use disorder

**Table 3:** Logistic regression predicting documented suicide attempt among veterans with an Indicator of unemployment, months 1–6 (*N* = 346,556)

Variable	aOR	95% CI		*p*-value
Sex Female (ref male)	0.90	0.80	1.02	0.100
Age (ref 45–54)				
18–34	**1.27**	**1.14**	**1.41**	**< 0.001**
35–44	**0.83**	**0.74**	**0.93**	**0.01**
55–64	**0.72**	**0.64**	**0.82**	**< 0.001**
65+	**0.41**	**0.34**	**0.50**	**< 0.001**
Race (ref Black)				
White	**1.69**	**1.54**	**1.86**	**< 0.001**
Another racial category	**1.64**	**1.36**	**1.99**	**< 0.001**
Missing/unknown	**1.59**	**1.30**	**1.95**	**< 0.001**
Ethnicity (ref Hispanic)				
Non-Hispanic	1.05	0.91	1.20	0.510
Unknown	0.84	0.64	1.11	0.220
Marital status (ref married)				
Unmarried	0.92	0.85	1.00	0.050
Unknown	0.81	0.57	1.16	0.260
Eligibility Category (ref SC > = 50%)				
SC < 50%	**0.81**	**0.73**	**0.90**	**< 0.001**
Not SC	**0.86**	**0.78**	**0.95**	**0.003**
Not SC, VA Pension	1.06	0.89	1.26	0.520
Other	0.85	0.58	1.24	0.390
Combat (ref none)	0.94	0.85	1.04	0.230
Military sexual trauma (ref none)	**1.38**	**1.24**	**1.53**	**< 0.001**
Rural (ref urban)	0.94	0.86	1.02	0.150
Region (ref West)	0.93	0.84	1.04	0.200
Midwest	**0.79**	**0.69**	**0.90**	**0.004**
Northeast	0.96	0.88	1.06	0.410
South	0.94	0.86	1.02	0.150
Community poverty level (ref low)				
High	0.97	0.84	1.12	0.680
Medium	0.96	0.85	1.09	0.570
Community education level (ref low)				
High	1.01	0.93	1.09	0.910
Medium	1.01	0.93	1.09	0.910
Missing	1.01	0.93	1.09	0.910
Inpatient admission				
Medical (ref none)	**1.21**	**1.06**	**1.39**	**0.010**
Mental health (ref none)	**1.46**	**1.30**	**1.63**	**< 0.001**
SUD (ref none)	**1.50**	**1.34**	**1.67**	**< 0.001**
Emergency department visit	**1.41**	**1.30**	**1.54**	**< 0.001**
Outpatient visits				
Primary care (n) (ref none)	0.99	0.98	1.004	0.150
Medical (n) (ref none)	**0.99**	**0.99**	**1.00**	**< 0.001**
Mental health (n) (ref none)	**1.01**	**1.00**	**1.01**	**0.000**
SUD (Any) (ref none)	**1.00**	**0.99**	**1.00**	**0.00**
Any medical diagnosis (ref none)	1.01	0.93	1.09	0.91
Any mental health diagnosis (ref none)	**1.63**	**1.45**	**1.84**	**< 0.001**
Any SUD diagnosis (ref none)	**1.43**	**1.31**	**1.57**	**< 0.001**
Baseline documented suicide ideation (ref none)	**2.04**	**1.86**	**2.23**	**< 0.001**
Baseline documented suicide attempt (ref none)	**3.12**	**2.87**	**3.40**	**< 0.001**
Documented suicide ideation (ref none)	**1.99**	**1.81**	**2.18**	**< 0.001**
Unemployment services use (ref none)				
High	1.02	0.94	1.11	0.700
Low	0.98	0.89	1.09	0.760
Homeless Services Use (ref N/A, No housing instability)				
High	**1.25**	**1.15**	**1.35**	**< 0.001**
Low	**1.32**	**1.14**	**1.53**	**0.003**
None	**1.16**	**1.03**	**1.32**	**0.020**
Justice Services Use (ref N/A, No justice involvement)				
High	0.99	0.88	1.12	0.900
Low	**1.34**	**1.15**	**1.56**	**0.002**
None	1.07	0.95	1.21	0.250

Notes. Model also controls for person month, calendar month, and calendar year. aOR = adjusted odds ratio; CI = confidence interval; SC = service-connected; SUD = substance use disorder

**Table 4:** Logistic regression predicting documented suicide attempt among veterans with an Indicator of justice involvement, months 1–6 (*N* = 200,487)

Effect	aOR	95% CI		*p*-value
Sex Female (ref male)	0.94	0.79	1.11	0.430
Age (ref 45–54)				
18–34	**1.19**	**1.05**	**1.35**	**0.010**
35–44	0.93	0.81	1.07	0.310
55–64	**0.76**	**0.65**	**0.89**	**0.001**
65+	**0.40**	**0.31**	**0.51**	**< 0.001**
Race (ref Black)				
White	**1.69**	**1.50**	**1.91**	**< 0.001**
Another racial category	**1.51**	**1.17**	**1.94**	**0.001**
Missing/unknown	**1.57**	**1.22**	**2.02**	**0.001**
Ethnicity (ref Hispanic)				
Non-Hispanic	**1.25**	**1.05**	**1.49**	**0.010**
Unknown	1.22	0.89	1.68	0.210
Marital status (ref married)				
Unmarried	0.97	0.87	1.07	0.500
Unknown	0.94	0.64	1.39	0.770
Eligibility category ( ref SC > = 50%)				
SC < 50%	**0.77**	**0.68**	**0.88**	**0.001**
Not SC	**0.81**	**0.71**	**0.91**	**0.006**
Not SC, VA Pension	0.94	0.75	1.20	0.630
Other	0.99	0.61	1.60	0.950
Combat (ref none)	0.91	0.81	1.02	0.110
Military sexual trauma (ref none)	**1.36**	**1.19**	**1.55**	**< 0.001**
Rural (ref urban)	1.00	0.90	1.10	0.940
Region (ref West)	1.08	0.95	1.23	0.230
Midwest	0.96	0.83	1.12	0.640
Northeast	0.95	0.85	1.07	0.410
South	1.00	0.90	1.10	0.940
Community poverty level (ref low)				
High	0.99	0.83	1.19	0.940
Medium	0.94	0.80	1.09	0.380
Community education level (ref low)				
High	1.11	0.95	1.29	0.210
Medium	1.04	0.93	1.17	0.500
Missing	**1.16**	**1.00**	**1.35**	**0.049**
Inpatient Admission				
Medical (ref none)	**1.30**	**1.09**	**1.55**	**0.003**
Mental Health (ref none)	**1.21**	**1.06**	**1.39**	**0.010**
SUD (ref none)	**1.15**	**1.01**	**1.31**	**0.040**
Emergency department visit	1.11	1.00	1.24	0.060
Outpatient visits				
Primary care (n) (ref none)	**0.98**	**0.96**	**0.99**	**0.030**
Medical (n) (ref none)	1.00	0.99	1.00	0.090
Mental health (n) (ref none)	1.001	0.997	1.006	0.550
SUD (Any) (ref none)	**1.00**	**0.99**	**1.00**	**0.048**
Any medical diagnosis (ref none)	1.02	0.92	1.13	0.680
Any mental health diagnosis (ref none)	**1.75**	**1.51**	**2.02**	**< 0.001**
Any SUD diagnosis (ref none)	**1.34**	**1.19**	**1.50**	**< 0.001**
Baseline documented suicide ideation (ref none)	**1.61**	**1.44**	**1.80**	**< 0.001**
Baseline documented suicide attempt (ref none)	**3.24**	**2.93**	**3.59**	**< 0.001**
Documented suicide ideation (ref none)	**4.01**	**3.57**	**4.49**	**< 0.001**
Justice services use (ref none)				
High	**1.24**	**1.12**	**1.37**	**< 0.001**
Low	1.09	0.98	1.23	0.120
Homeless services use (ref N/A, No housing instability)				
High	**1.42**	**1.28**	**1.58**	**< 0.001**
Low	**1.37**	**1.14**	**1.64**	**0.001**
None	**1.21**	**1.04**	**1.40**	**0.010**
Employment services use (ref N/A, No unemployment)				
High	1.09	0.95	1.26	0.210
Low	0.88	0.72	1.08	0.220
None	0.98	0.88	1.10	0.750

Notes. Model also controls for person month, calendar month, and calendar year. aOR = adjusted odds ratio; CI = confidence interval; SC = service-connected; SUD = substance use disorder

Across all three study cohorts, several correlates were consistently associated with risk of documented suicide attempt during the 6 months following identification into the cohort. Specifically, younger age (versus the 45–54 age group) and White race (versus Black/African American race) were associated with greater odds of documented suicide attempt. MST was positively and consistently associated with incident documented suicide attempt among all three cohorts (i.e., housing instability cohort: aOR 1.44, 95% CI:1.32–1.57; unemployment cohort: aOR 1.38, 95% CI:1.24–1.53; justice involvement cohort: aOR 1.36, 95% CI:1.19–1.55). Other medical factors that were consistently associated with greater odds of documented suicide attempt included inpatient admission, any mental health diagnosis, and any SUD diagnosis.

## Discussion

In this retrospective longitudinal study using a secondary analysis of VA administrative data, the results partially supported the hypotheses that utilization of any of three types of social services would be positively associated with documented suicide attempt. The interpretation of these results requires careful attention to contextual perspectives of pragmatic studies alongside limitations of methods and data availability. For example, among the cohort of patients with an indicator of housing instability, high utilization of justice-related programs was associated with a 24% increase in odds of suicide attempt compared to patients with housing instability who did not use justice services. These findings should not be interpreted as social service use causing suicide attempt. First, as emphasized in the methods, the outcome is referred to as documented suicide attempt because the data were gathered from administrative records and not individual patient self-reported suicide attempt. More plausibly, patients experiencing social needs may have greater distress [38], and that greater distress is associated with both suicide risk and more frequent social services, which begets greater opportunities to detect suicide attempt. A further example of this is exemplified in the positive association of inpatient care with suicide attempt documentation. Although attempts were made to control for greater distress through available EHR data (e.g., mental health service utilization), ultimately, proximal measures of self-reported patient distress were not available in the EHR data.

The findings for social service program use may inform increased efforts of health system-based suicide detection and prevention. Even after adjusting for numerous factors, including mental health diagnoses and mental health treatment, utilization of social services remained significantly and independently associated with documentation of suicide attempt. Health systems are quickly moving to develop and deploy predictive analytics for suicide prevention [39], and it is unclear if those predictive algorithms include social service utilization variables. No other healthcare system in the US has developed and implemented as many mental health and suicide prevention initiatives as the VA [40]. Consequently, because suicide prevention is woven through the VA system, VA social service programs promote suicide risk awareness (e.g., screening, training about warning signs); [41] for example, permanent supportive housing programs include intensive case management to evaluate, procure, and establish a patient into housing [42].

Social service utilization could have a two-fold effect on the present findings: (1) patients facing adverse social determinants had greater distress that increased likelihood of suicidal thoughts and behaviors, and (2) use of social services increased detection of suicide risk. A prior suicide attempt is one of the strongest risk factors for future suicide attempts and for dying by suicide [16], so it is crucial to document suicide risk in the EHR to facilitate intervention with these high-risk patients. The present results support that social services are one such arena for this documentation. Although the present results identified independent associations for social services utilization, further research is needed to develop and test different study designs within healthcare settings to evaluate outcomes of social services utilization. These may include designs that can recruit patients seeking social services in prospective studies with self-reported data about mental health and suicidality in addition to administrative data.

The study also uncovered several instances in which service utilization was not significantly associated with the outcome, namely unemployment services. There are at least three potential explanations. First, it is possible that, of the three adverse social determinants incorporated in this study, unemployment may be less severe for mental distress compared with impending or current housing instability or legal problems. For example, veterans are more likely to be married than nonveterans [43], which could suggest a greater potential for dual-earners in a household that buffers financial stress related to unemployment. Second, veterans utilizing unemployment services could be experiencing better mental health in preparation to seeking or rejoining the workforce, with suicide attempt less likely to be detected or documented by providers of unemployment services. Third, patients in the present study may have accessed non-VA services for adverse social factors either in addition to or in lieu of VA social services. Unfortunately, VA does not systematically collect data about use of non-VA social service programs, so it was not possible to account for this potential effect. VA has more recently begun covering non-VA community care; [44] however, this effort has been focused on healthcare. As the US enhances focus on social care as integral to healthcare [45], future efforts must better understand how VA patients may utilize non-VA social service programs.

Several other limitations must be noted. As a retrospective study of extant administrative data, there was inability to control for potential biases around which patients utilized services (i.e., non-randomization). Although the VA has national data systems to document and track suicide-related phenomena [4], it is possible that detection of suicide attempt is underreported, as patients may not disclose an attempt to a provider [46–48], and evidence of the attempt may not be recorded in administrative records. In addition, the data could not account for quality of services received, only *that* services were received. Quality of social services, much like quality of health care, may be a potent determinant of patient disclosure of suicidal thoughts and behaviors.

In our findings, more frequent receipt of social services for housing instability, justice involvement, and unemployment were positively associated with documented suicide attempt, and thus highlight opportunities to identify and intervene with patients at high risk of future suicide attempt or death from suicide. The results also bolster the relevance of social service utilization in understanding detection in EHR of suicide attempt among veterans receiving care from the VA. Because social services utilization signals more frequent engagement with the VA health system, social service providers are as important to suicide detection and prevention as other health care providers. There is increasing emphasis on targeting upstream, nonmedical factors as points for suicide prevention [49], and further research is needed to better understand how social service providers working in housing, employment, and legal assistance can be vital points of intervention, especially for patients experiencing adverse social determinants of health.
